# Survey on Impact of Operational Policies and Procedures on Patient Satisfaction at a Rural Free Healthcare Clinic in Florida

**DOI:** 10.7759/cureus.11730

**Published:** 2020-11-27

**Authors:** Rachel D Truong, Nathan Kostick, David Vu, Lily Y Chen, Elliott Cheung, Nadine Dexter

**Affiliations:** 1 Medicine, University of Central Florida College of Medicine, Orlando, USA

**Keywords:** rural healthcare, free clinic, patient safety, patient satisfaction, quality improvement

## Abstract

Aims

Free healthcare clinics provide highly necessary services for the populations they serve, particularly in rural, low socioeconomic areas. When assessing for quality of clinic performance, it is important to consider the background of the population it serves in addition to observations given by clinic volunteers. Contextualizing the healthcare challenges patients face will help the clinic assist them to a greater capacity. Here, we assess how different areas of clinic operations (service, safety, accessibility, interactions with volunteers, and wait time) impact patient satisfaction in the setting of a small, rural, free clinic.

Methods

Eligible participants were asked to fill out an anonymous, 21-question survey that assessed their experiences and perspectives on various aspects of the clinic. The study was single-blinded with clinic staff unaware of the nature of the study.

Results

Thirty-five patients responded to the survey. Overall, patients were extremely satisfied with the clinic with an average Likert score of 4.8/5; 14 of 15 categories scored a four or higher. Wait time scored lowest (3.6/5), with waits up to eight hours. Additionally, we found that transportation was not a major barrier to patients, with 80% arriving by personal transport.

Conclusions

The clinic provided valued and satisfactory services without coming across as discriminatory to the community. Areas of improvement include wait times, role clarification, and better integration of medical students. Additional studies to further understand the community will facilitate tailoring healthcare to a rural underserved population in the Southeastern United States.

## Introduction

Free clinics play a key role in ensuring access to healthcare among the uninsured patient population. A 2010 survey estimated that of over 3.1 million medical visits at free clinics, 92.2 percent of patients were uninsured [1]. States without Medicaid expansion contain a majority of clinics recognized by the National Association of Free and Charitable Clinics (NAFC), suggesting a continued necessity of these facilities in medically underserved communities [2]. Access to care continues to be a significant barrier despite the availability of free clinics, which impedes the clinics' ability to reduce health disparities across socioeconomic levels [3]. For instance, free clinic patients, especially rural populations, may encounter transportation issues and perceive a lack of quality healthcare [4,5]. A variety of factors, which can vary based on region [4] and the patient population being studied, contribute to these obstacles. It is therefore necessary for free clinics to survey their patient populations to identify their own shortcomings and implement effective solutions for their patients.

The St. Thomas Aquinas Free Medical Clinic (STA) operates under the Catholic Charities of Central Florida and the St. Thomas Aquinas Catholic Church in St. Cloud Florida. It provides access to free healthcare services such as preventative screenings, health education, case management, and referrals to specialists or social services. Clinic patients are without health insurance and with income no higher than 200% above the poverty line in Osceola County. In contrast, the clinic is predominantly staffed by volunteers from various health centers around the area, many of whom do not share the same backgrounds as patients at the clinic. As such, there exists a need to contextualize the challenges that patients face and identify specific factors that influence perceptions of the clinic and its staff.

Patient satisfaction is a widely used indicator of clinic performance [6,7] and has been linked to improved retention [8] and outcomes in chronic care [9]. The current model of assessing patient satisfaction compares a patient’s perception of their clinic experience with their prior expectations. This is subject to numerous confounders such as financial limitations of the patients or their clinics, socio-cultural differences, and their personal health outcomes. Here, a patient satisfaction study was completed to dissect and understand in context patients’ perceptions of their experiences at the clinic.

## Materials and methods

Every Wednesday, STA clinic opens at 3:30 PM for patient intake. The first of the patients is seen at 5:30, and the clinic day typically concludes at 8 PM. At the front desk, the clinic manager selects 14 patient cases and advises the remaining potential patients to return the following week due to capacity. The patient group seen consist of the most urgent cases and/or the ones who were turned away the prior week. Patients are assigned at random with some examined by a medical student prior to seeing a provider, after which they are sent to discharge. The 14 patients chosen to be seen that day were recruited to complete the survey at the end of their visit.

Study participants

Participants were recruited from the Saint Thomas Aquinas Free Medical Clinic. Eligible subjects were 18 or older and qualified for general medical care offered at the Saint Thomas Aquinas Free Medical Clinic (ie lacked insurance, below 200% the poverty line, and resident of Osceola county). Participants were excluded if they visited the clinic during a specialty care day (ie orthopedics or cardiology), were turned away on a normal clinic day, had already completed the survey, or were unable to read English or Spanish. It has been noted that no patients were excluded from this study due to a language barrier, as all patients seen over the course of this study were literate in either English or Spanish.

To ensure that patients who were seen repeatedly over this period completed the survey only once, they were asked if they had completed the survey before. If the patient responded that they had completed the survey before, they were not asked to complete it a second time. 

Throughout the duration of this study, an average of 20 patients would arrive at the clinic per operating day and six would be turned away. Over the study period, the clinic saw 56 patient cases. Two patients declined due to having taken the survey before and 19 declined participation in the study. A cohort of 35 participants consented and completed the survey.

Study design

**Figure 1:**
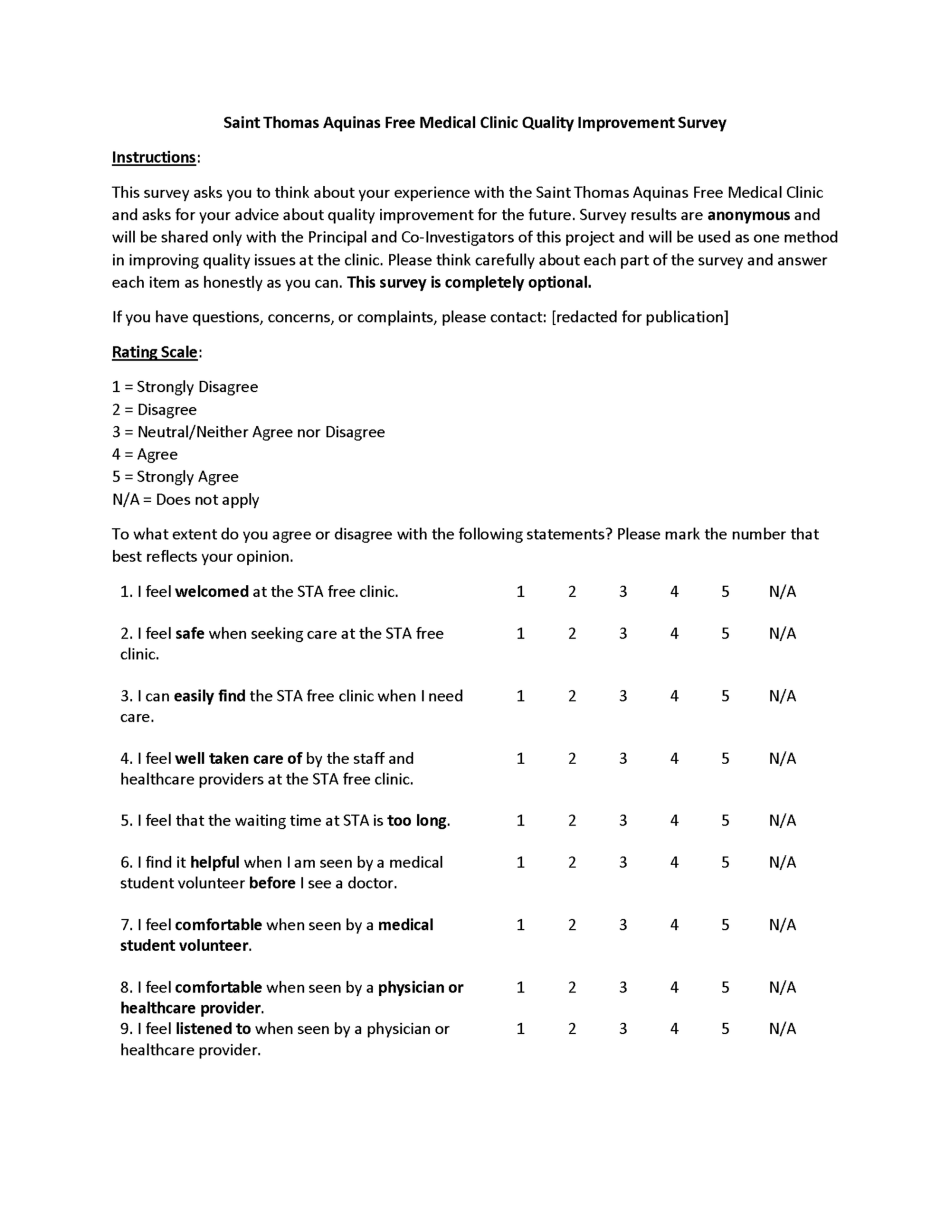
Sample survey page 1

**Figure 2:**
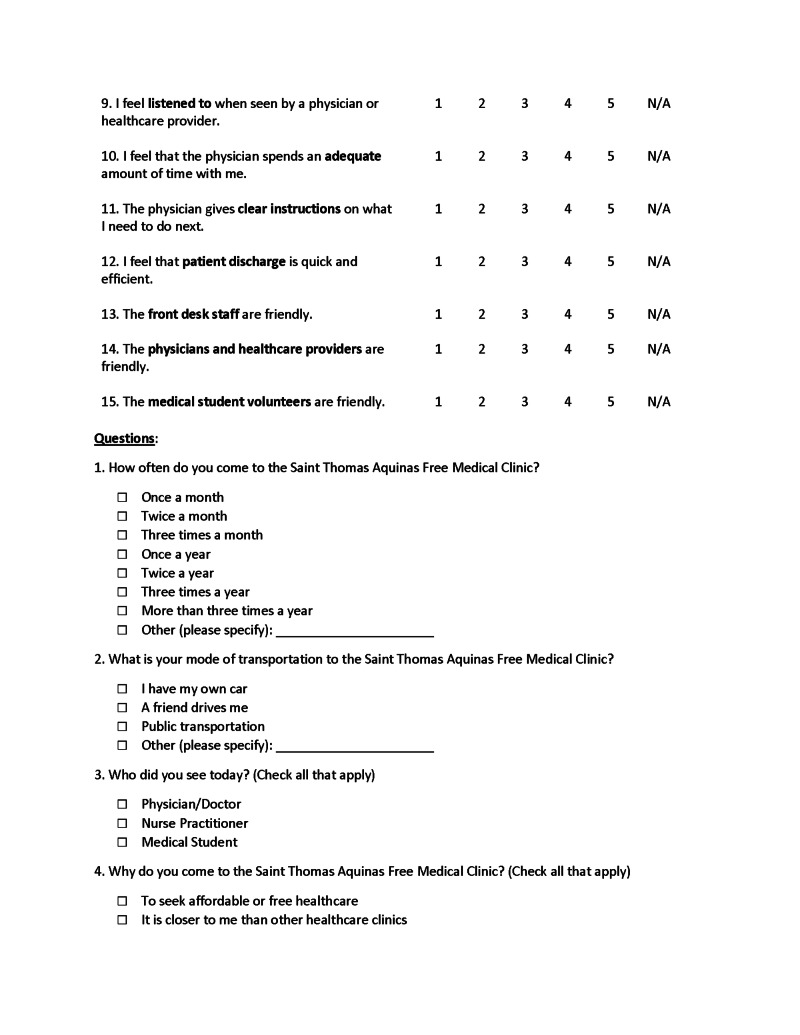
Sample survey page 2

**Figure 3:**
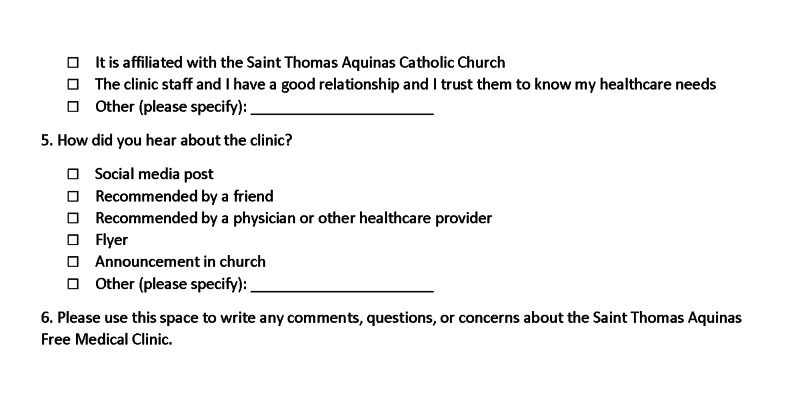
Sample survey page 3

Patients responded to a one-time paper survey in either English or Spanish at the end of their visit at a single location. Anonymous surveys were completed and submitted away from any supervision, given a sequential number for organizing only, and digitized by blinded researchers. Twenty-one questions assessed the cohorts’ perceptions of the clinic and allowed for patients to rate their experiences under specific categories. The first 15 questions of the survey asked participants to rank various aspects of their clinic experience (attitude of healthcare professional, ease of finding the clinic, quality of service, etc.) on a 5-point Likert scale from 1 for Strongly Disagree to 5 for Strongly Agree. Five more questions were optional checkbox questions asking patients about their frequency of visitation, method of transportation, what medical care provider(s) they encountered during their visit, and how they found out about the clinic. The final question was an additional free-response space for comments. A sample copy of the survey is shown in Figures 1-3. For patients who were visiting the clinic for the first or second time, they were instructed to check "other" and note that fact in the provided line.

Data was collected between early-October and late-December of 2019. Investigators conducted the survey during four clinic sessions throughout this period. 

Data analysis

Sample size for the purposes of statistical analysis was established as n = 35 based on the number of participants who completed the study. Mean scores and standard deviations were then calculated based on the Likert scale point system. The checkbox questions were analyzed for distributions of participant selection. Data recording, statistical analysis, and data visualization were completed in Microsoft Excel.

## Results

**Figure 4:**
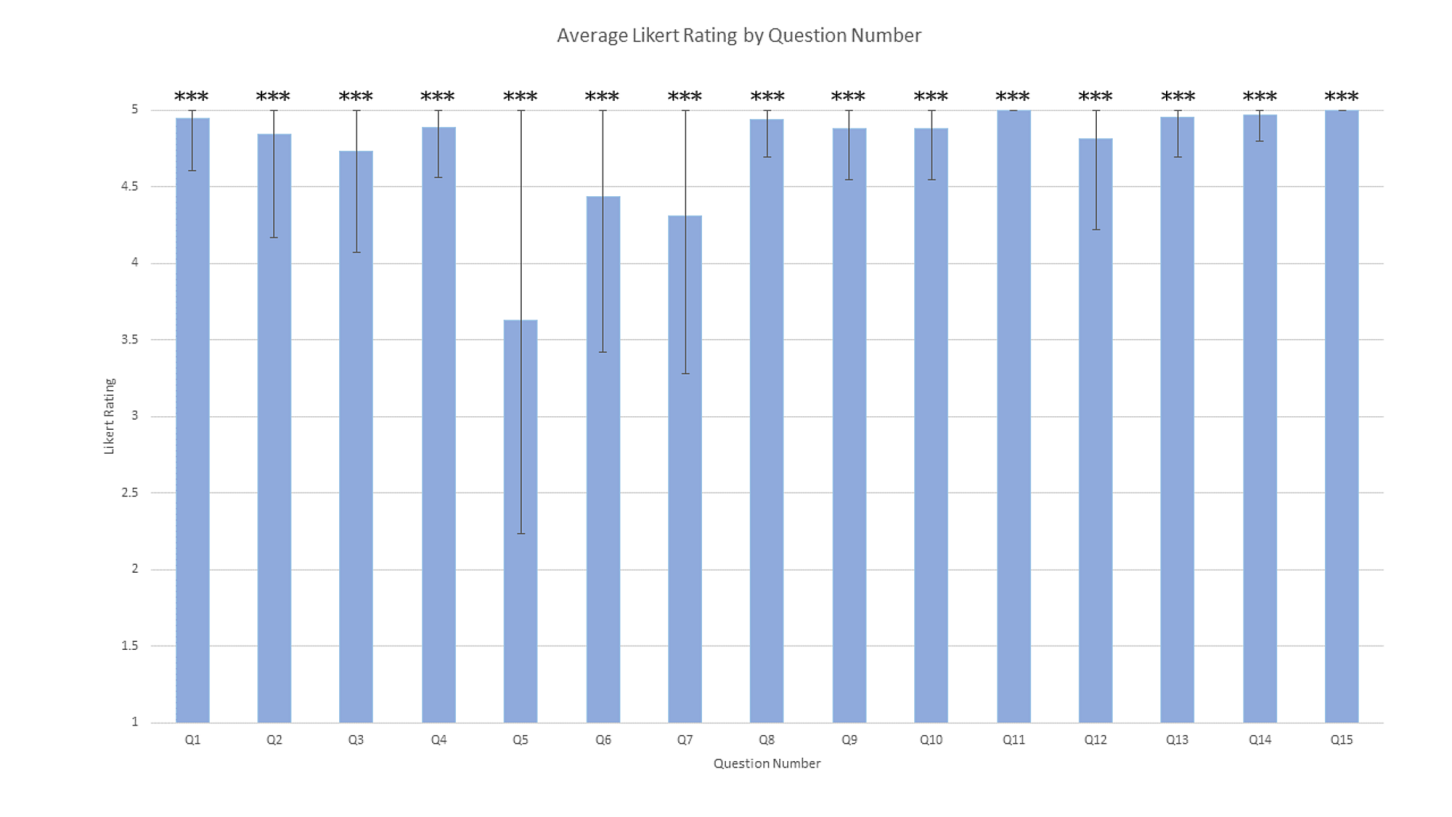
Average Likert scores for survey questions. Bar graph depicting average Likert scores and standard deviations of the first 15 survey questions. The three asterisks above each error bar represents standard deviation plus mean that crosses the maximum value, (ie. >5)

**Figure 5:**
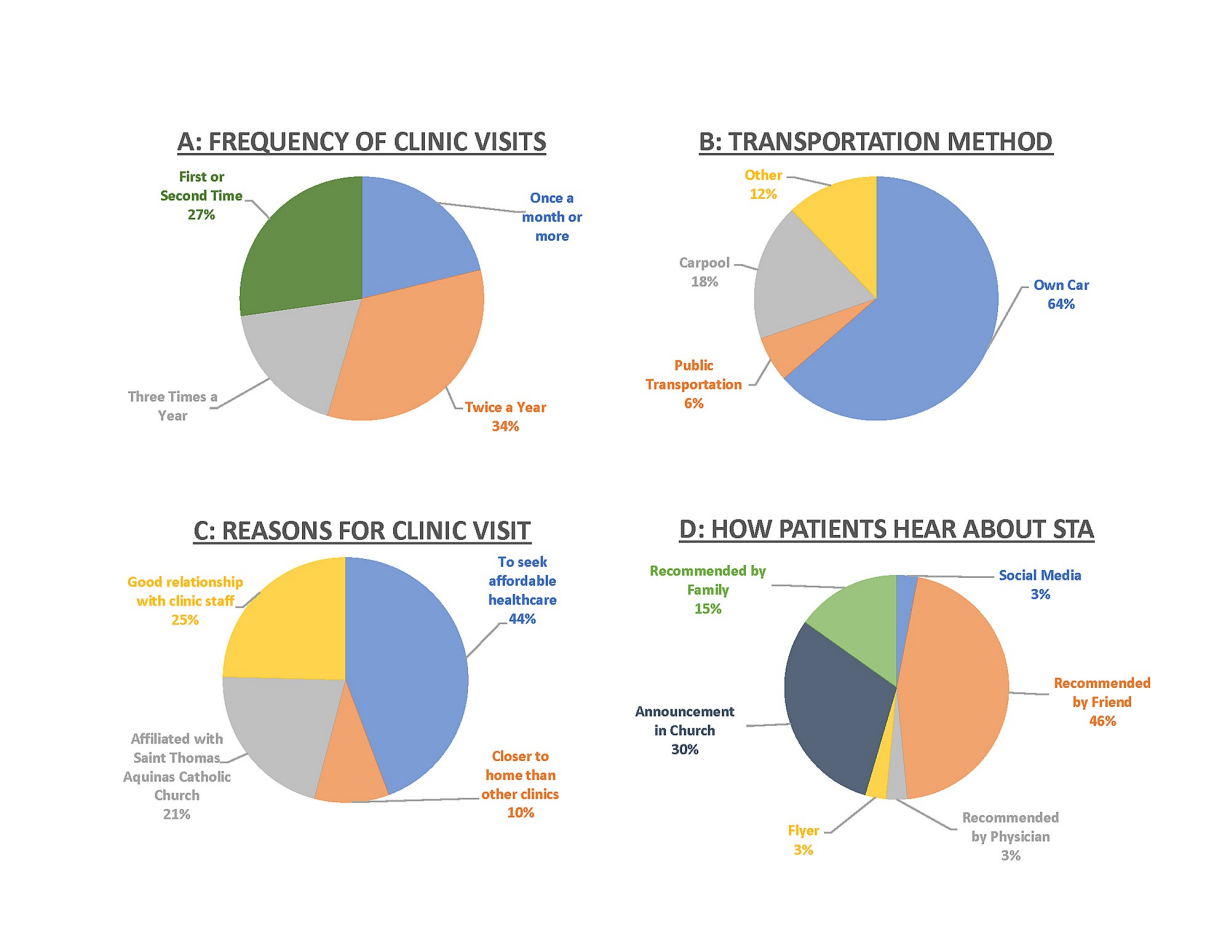
Pie charts of responses to four of the five optional checkbox questions. The responses to the fifth question (“Who did you see today? Check all that apply.”) were affected by patient confusion over role definitions and do not convey useful information in the form of a pie chart.

Of the 35 patient surveys, 20 surveys (57%) were submitted in Spanish and 15 were submitted in English. All participants were residents of Osceola County with family incomes less than 200% below the poverty line. Overall the patient satisfaction was high with the average of the 15 Likert scale questions of 4.8/5 wherein 1 indicates least satisfied and 5 indicates most satisfied. The lowest scoring section was regarding the wait time, with 40% selecting 3 or less and an overall 3.6 average on the Likert scale. We found that a significant number of patients do not know who is treating them: 30% of respondents reported being seen by a combination of clinicians that STA clinic does not offer (ie both an Nurse Practitioner (NP) and a Medical Doctor (MD) or a Medical Student, NP and MD). Other items of note: 80% of patients arrived by their own vehicle or a vehicle driven by a friend/family member. STA patients surveyed were almost exclusively established patients, with less than 20% of patients being first- or second-time patients and 67% going twice per year or more frequently. The primary reason cited for choosing STA was for affordable medical care, followed by having a pre-existent physician-patient relationship and its association with the STA church. The survey results are represented in further detail in Table 1, Figure 4 and Figures 5A-5D.

**Table 1:** Results of Likert scale-based survey Descriptive statistics summarizing the results of the  20 question Likert scale-based survey, which used a range of 1 to 5. (1 = Strongly Disagree, 2 = Disagree, 3 = Neutral, 4 = Agree, 5 = Strongly Agree)

Perception of Clinic Visit	Median	Average Score	Standard Deviation
While visiting Saint Thomas Aquinas, I feel…			
… welcomed.	5	4.92	0.34
… safe.	5	4.80	0.70
… well taken care of.	5	4.90	0.30
... it is easy to find.	5	4.74	0.67
If I see a medical student volunteer before seeing a doctor, I feel…			
… that it is helpful.	5	4.40	1.0
… comfortable.	5	4.31	1.03
… that they are friendly.	5	5.0	0
When I see my healthcare professional at Saint Thomas Aquinas, I feel…			
… comfortable.	5	4.94	0.24
… listened to.	5	4.90	0.30
… adequately cared for.	5	4.90	0.30
… clear on what to do next.	5	5.0	0
… that they are friendly.	5	4.97	0.17
At Saint Thomas Aquinas, I feel that…			
… patient discharge is quick and efficient.	5	4.81	0.59
… the front desk staff is friendly.	5	4.95	0.26
... the wait time is reasonable.	4	3.60	1.40

## Discussion

This study utilizes measures of patient satisfaction to better understand the patients seen at STA and identify areas for improvement at an interprofessional rural free clinic. While a wealth of research on facets of free clinics has been published, the body of literature specifically on rural free clinics is paltry in comparison, in part due to the scarcity of providers despite the significant need for and promotion of rural healthcare in the United States [10,11]. However, patient satisfaction has demonstrated to be pertinent to patient compliance and outcome [12,13], so improving healthcare delivery at a rural Florida free clinic necessitates understanding the perspectives and opinions of the community which it serves.

Because the surveys in this study were completed and submitted away from any supervision and digitized by blinded researchers, the responses most likely are genuine feedback. The 35 unique patient responses provide several points of interest.

Differences in average scores were marginal between most (12 of 15) statements (Figure 4). Patients found all volunteers to be very approachable. A strong perception of friendliness is imperative when various differences in background between providers and patients can potentially lead to worse patient evaluations of care [14,15]. Furthermore, patients ubiquitously found physician instructions to be clear (Table 1). This demonstrates respectful communication of the physician instructions despite potential differences in culture and language. No inferences can be made regarding provider-patient language pairings nor interpretation services since all surveys were anonymized. Nevertheless, patients who feel comfortable in communication with providers are more likely to comply and experience good outcomes [16,17], so the clinic will continue utilizing and refining existing communication methods.

It is also worthwhile to comment on the significance of the overall high satisfaction: the perception of discrimination itself is quantifiably detrimental to clinical outcomes [18,19]. Here, the various positive impressions related to metrics like those used by Piette et al. are suggestive of reduced perception of discrimination [18]. Furthermore, patients are expressing a sense of accessibility of and willingness to access services at the clinic. This satisfaction is critical to the further development of the clinic as well, since it suggests potential avenues of deepening relations with the community [18] and reducing delays in seeking needed medical attention [20].

Only three statements out of the 15 assessed by Likert scales received average ratings below 4.8; this distinction emphasizes the general areas of improvement needed at the clinic. Based on the survey, the least satisfaction was seen in responses to wait times (Table 1). Official statistics on wait times have not been recorded at the clinic, but front office staff recall wait times up to eight hours due to higher volumes of complex cases, which has sometimes caused the clinic to be open well past its closing time of 8 PM. Existing literature varies in stances on the correlation of wait times to patient satisfaction in outpatient clinic settings [9,21,22]. We found that our patients felt well taken care of by the clinic staff despite long wait times (Table 1). Regardless, the contrast between this result and the others polled indicate that the patients have noticed the lengthy wait, so we will explore methods of increasing efficiency [23] and then alternative options for increasing engagement [24].

The remaining two points of improvement focus upon the presence and actions of the medical student. While patients ubiquitously view medical students as friendly (Table 1), they are less comfortable with seeing medical students than physicians and nurse practitioners. While the survey cannot discern the specific reasons for discomfort, the combined low ratings for the statements describing comfort and perception of helpfulness when seen by a medical student suggests that patients are not as concerned about student professionalism as they are uncertain over the standard of care received. We thus will seek to identify specific points of concern related to medical students because multiple sources of concern could stem from the clinic system: at the clinic, patients are made aware of the presence of medical students, but a random fraction of patients are interviewed and physically examined by students before a physician or nurse practitioner enters the room. In short, while medical student participation in free clinics benefits students [25] and provides useful resources [26], meticulous, evidence-based steps should be taken to confirm willingness, provide reassurance, and ensure excellent care to patients who encounter medical students during their visit.

The remaining questions yielded a series of observations and inferences regarding the satisfaction of the patient population seen at STA, as shown in Figures 5A-5D. Most patients who completed the survey were established patients. The regularity of patients may have one or more of several causes that should be scrutinized, including the quality of healthcare delivered and accessibility relative to other free clinics. New patients could have also declined to participate in the survey due to miscellaneous reasons.

Additionally, the majority of new patients come to the clinic by word of mouth. Despite the minimum budgeting put into advertising, the clinic reaches full capacity every week. Based on attendance, we appear not to need an immediate increase in advertising.

Only 18% of surveyed patients did not own or have access to a car. For the existing patient population, access to care does not warrant the introduction of a transportation service. However, when we have the capability to expand operations, we will revisit this topic. With the integration of telemedicine that occurred after the end of data collection for this study, it is possible that new patients who previously could not physically access the clinic will receive necessary medical attention. The potential shift in patient population is thus an important point for future studies at the clinic, as the new technology will further elucidate any unseen needs for clinic-partnered transportation arrangements.

The cohort surveyed also demonstrated that there is confusion in the community between provider types. Many patients do not know whether they are being seen by a Nurse Practitioner (NP) or a Medical Doctor (MD). The immediate implications of this confusion are unclear, but patients may benefit from having more certainty and confidence in understanding the different aspects of their care as well as establishing unambiguous role expectations in the continuation of their care [27].

Limitations

Although front desk staff are consistent, physicians, healthcare providers, and medical students volunteer in rotation from a consistent pool of participants. It is difficult to discern the performance of individuals from this survey, even though the strong positive impressions overall are promising. The survey was also cross-sectional, but tracking perceptions longitudinally would provide insight as to the effectiveness of any changes implemented. Additionally, only a subset of patients who attend the clinic could be surveyed, and therefore, the findings may not be representative of the community as a whole.

Furthermore, additional patients should be surveyed for a more comprehensive and accurate understanding of community perceptions. The information gathered during the surveying process should be expanded as well to include demographics because cultural differences may influence patient perceptions towards the clinic experience [28]. In a county estimated to be 55.8% Hispanic or Latino and 79.1% White [29], it is important to gather information on cultural and ethnic backgrounds of patients who provide feedback. Differences in experiences between English and non-English speakers should be assessed as well to adjust for confounding factors due potentially to language barriers. However, it should be noted that many volunteers speak Spanish at least at a conversational level, which may reduce quality disparity, but the comparison should be studied further.

## Conclusions

In conclusion, the community we serve values the services we provide and does not feel discriminated against at the clinic. Dedicated training programs to improve interpersonal relationship skills are thus currently not necessary at STA, and improvement efforts may be focused elsewhere. Despite the statistically overriding assertion of satisfaction by patients, more steps are needed to improve wait times and clarify different roles as well as address the discomfort patients may have with medical students. As STA continues to evolve and grow, further surveys and quality improvement projects will be implemented to better understand and serve the rural Central Floridian community and provide an even more comprehensive example for small rural free clinics that can be established in the region.
